# Analysis of supplemental wildlife feeding in Mississippi and environmental gastrointestinal parasite load

**DOI:** 10.3389/fvets.2022.995437

**Published:** 2022-09-26

**Authors:** Miranda H. J. Huang, Steve Demarais, W. Cooper Brookshire, Bronson K. Strickland

**Affiliations:** ^1^Department of Wildlife, Fisheries and Aquaculture, Mississippi State University, Mississippi State, MS, United States; ^2^Department of Clinical Sciences, College of Veterinary Medicine, Mississippi State University, Mississippi State, MS, United States

**Keywords:** *Baylisascaris procyonis*, coccidia, *Cryptosporidium*, deer, gastrointestinal parasites, *Giardia*, supplemental feeding, nematodes

## Abstract

Wildlife species are host to a variety of gastrointestinal parasites (GIPs). Artificially concentrating animals may increase the risk of disease spread due to increased GIP load and associated environmental load. Supplemental feeding of deer is common among hunters and known to concentrate animals, but there is limited knowledge of how it affects GIP environmental load. GIP load was compared between ecologically-equivalent pairs of sites in Mississippi with and without year-round supplemental feeding (average distance between pairs = 147 m). During May-August in 2019 and 2020, feces from white-tailed deer and raccoons were collected and examined for the presence of nematodes, coccidia, *Giardia* spp., *Cryptosporidium* spp., and *Baylisascaris procyonis*. On average, fed sites had 8 more deer (241% increase) and 2 more raccoon fecal piles (540% increase) than unfed sites. Average parasite loads for individual fecal samples did not differ between fed and unfed sites, but the greater number of deer and raccoon fecal piles at fed sites (*p* < 0.0001) produced 231% and 308% greater environmental loads of nematodes and coccidia, respectively. Spin feeders, the only feeder type that distributed feed on the ground, had 326% more coccidia in feces on average compared to other feeder types (*p* < 0.03). These results show that supplemental feeding of white-tailed deer, especially with spin feeders, increases environmental loads of GIP and the potential for transmission of parasitic diseases.

## Introduction

Supplemental wildlife feeding is a popular management action for white-tailed deer, *Odocoileus virginianus*, (WTD) enthusiasts, practiced by 89% of hunters in Arkansas (1) and about half of Mississippi hunting clubs (W. McKinley, personal communication). Hunters and managers use feeding with various goals including increasing recreational opportunities, food resources during times of scarcity, and body condition (2). It is known to concentrate animals in a small area which can intensify otherwise insignificant infections (3). Anthropogenic food sources (e.g., supplemental feeding) have been implicated in the transmission of many wildlife diseases such as bovine tuberculosis in WTD (4), raccoon roundworm (*Baylisascaris procyonis*) in raccoons [*Procyon lotor*; (5, 6)], and endoparasites in wild boar [*Sus scrofa*; (7)].

Gastrointestinal parasites (GIPs) are notable due to their potential to infect many different species (8). Though many parasite genera or species are host-species specific (e.g., *Eimeria* spp.), others such as *Cryptosporidium parvum* and *B. procyonis* are not. *C. parvum* has reservoirs in humans and domestic animals and has been documented in a variety of wildlife species including WTD (9, 10). *B. procyonis* is a well-documented zoonotic threat, and it is transmitted by many animals that typically visit supplemental wildlife feeding stations [e.g., rodents, turkeys (*Meleagris gallopavo*), raccoons, and many others] (11–13).

Wildlife's contribution to landscape-level parasite contamination, and thus transmission risk, can be calculated as environmental load (14). Environmental load calculations quantify parasites by wildlife population or geographic area and may indicate potential for transfer, especially for fecal-oral transmitted species (15, 16). Though past research correlated wildlife GIPs and human-supplied food, there is little research directly comparing wildlife with and without feeding sites (17) or addressing how environmental load changes over time at long-term feeding sites.

To address these gaps in the literature, this study compared the environmental load of gastrointestinal parasites at paired fed and unfed sites. Eggs and oocysts were identified to genus level, and categorized as either “nematode” or “coccidia” based on microscopic morphology.

In previous fecal parasite prevalence studies in WTD, nematode eggs identified by morphology were consistent with a broad range of strongylid species in three super familes: Ancylostomatoidea, Strongyloidea, and Trichostrongyloidea (18). In WTD, coccidia oocytes identified by morphology were consistent with the *Eimeria* spp. (19). In previous fecal parasite prevalance studies in raccoons, nematode eggs identified by morphology were consistent with *Physaloptera rara* and *Gnathostoma procyonis*. Though not a nematode, *Macracanthorhynchus ingen* is common in raccoons in Mississippi, and the eggs have similar morphology to some raccoon nematodes. (6, 20–22). In raccoons, oocysts identified as coccidia were morphologically consistent with *Eimeria* spp. and *Sarcocystis* spp. (23).

Nematode eggs and coccidia oocytes from WTD and raccoon feces were chosen to estimate envirnomental load because of their high expected prevalance at supplemental WTD feeding sites, ease of identification with routine laboratory techniques, and likely increased transmission risk with increased envirnomental contamination. Coccidia species are transmitted by the fecal oral route (24), while many nematode species are transmitted through direct contact with larvae in the environment (25). Because of these transmission routes, it is likely that increased environmental loads are associated with increased transmission risk. Gastrointestinal parasite load was also assessed by feeder type and duration of feeding. Finally, feces collected from both fed and unfed sites were tested for *Giardia* spp., *Cryptosporidium* spp., and *B. procyonis*. It was hypothesized that environmental load of gastrointestinal parasites would be greater at fed sites compared to unfed sites (6, 7) as well as with longer duration of feeding compared to short-term feeding because WTD use of feeders increases over time (26).

## Materials and methods

**Figure 1:**
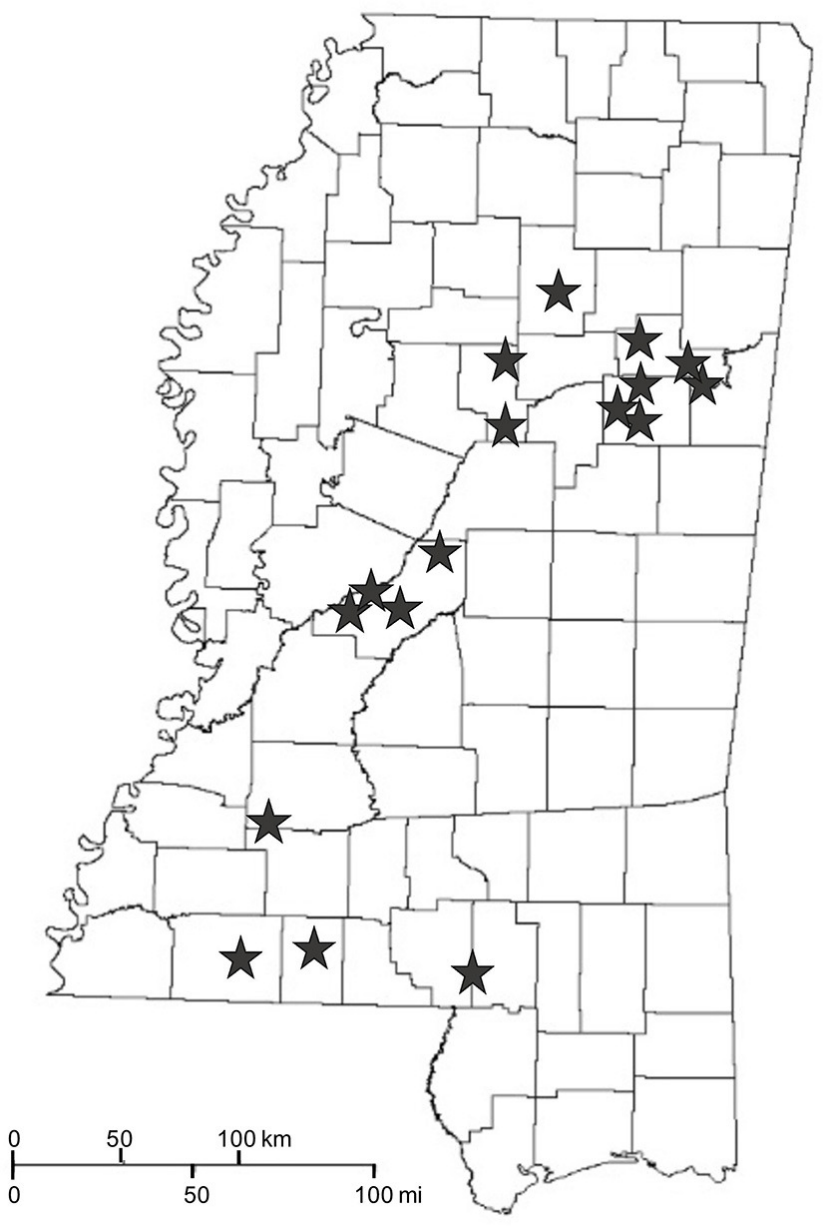
Properties (stars) sampled in Mississippi, USA during May-August of 2019 and 2020.

**Figure 2:**
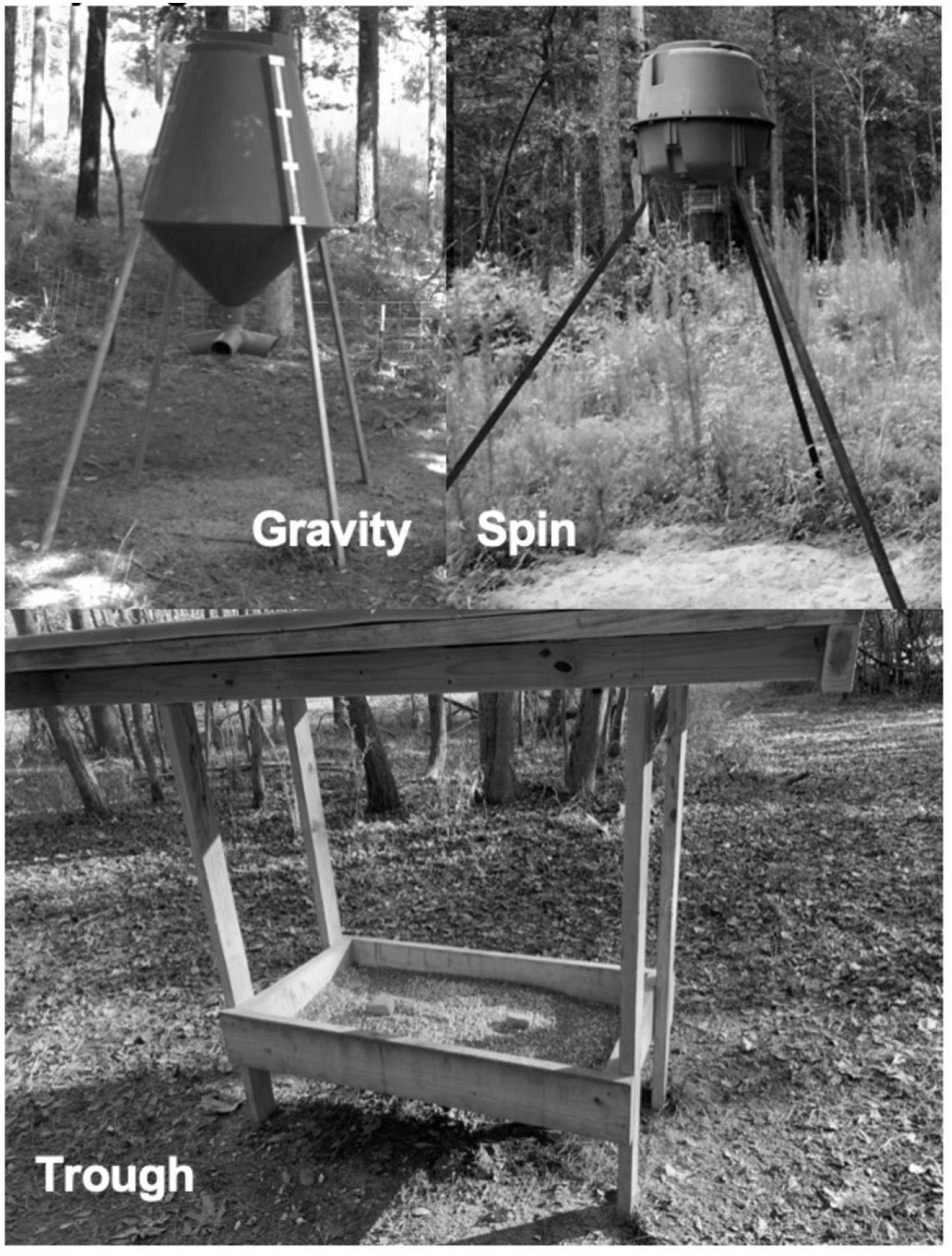
Feeder types (gravity, spin, and trough) sampled in Mississippi, USA during May-August of 2019 and 2020.

WTD feeders that were in year-round use for at least 1 year were sampled on 17 properties (Figure 1) in Mississippi, USA. Each feeder had been in year-round use for 1–10 years (mean ± SD; 3.9 ± 2.3 years). Feeding duration was categorized into short term (<5 years) and long term (≥5 years). The types of feeders included spin (*n* = 31), trough (*n* = 26), and gravity (*n* = 22; Figure 2). Most feeders were privately-owned and operated, so their placement was non-random.

Each fed site (i.e., area with a feeder) was paired with an ecologically-equivalent, unfed reference site. Reference sites were areas without feeders but with similar characteristics (e.g., plant community, prescribed fire regime, and proximity to bodies of water, agronomic food plantings, and infrastructure). Seventy-nine feeder sites had viable, ecologically-equivalent unfed pairs. Feeders and their reference sites were 147 ± 70 m apart (average ± SD). To match characteristics as closely as possible between fed and unfed sites, reference sites were chosen non-randomly.

Site pairs were established in a variety of vegetation types: closed canopy pines, thinned pines, and bottomland hardwoods. The pairs were in seven different soil resource regions: Blackland Prairie, Interior Flatlands, Lower Coastal Plain, Lower Thin Loess, Upper Coastal Plain, Upper Thick Loess, and Upper Thin Loess (27).

Visitation by animals and humans (researchers excluded) was monitored at each site using camera traps (Bushnell Trophy Cam HD Vital V3 Game Camera, Bushnell, Overland Park, Kansas, USA) placed 4–5 m from site center. Increased visitation around feeders was expected so cameras settings to reduce photo redundancy and double counting of individuals were chosen. A 25-min interval between trigger events was used with one photo per event and a scanning photo every hour. Species and number of individuals from each photo were recorded.

Sampling occurred on the same day for each pair of sites to limit the influence of factors beyond feeding status (e.g., seasonal differences, weather). From May to August of 2019 and 2020, feces were counted and collected along concentric, circular transects out to a 25 m radius from site center. Feces were identified by visual characteristics to species level (28) and samples deemed fresh enough for GIP counts (moist and soft to the touch) were collected. Samples were placed on ice in the field and refrigerated in the lab until processing, which occurred within 48 h of collection.

To quantify WTD and raccoon fecal parasites, the McMaster's technique was used to determine the number of nematode eggs per gram of feces (EPG) and coccidia oocytes per gram of feces (OPG) in all fresh WTD and raccoon feces (29). Non-quantitiave test tube flotation testing with Sheather's solution was performed on all raccoon feces collected to surveil for *B. procyonis*. All samples with eggs suggestive of possible *B. procyonis* were submitted for confirmation by RT-PCR to Zoologix (Chatsworth, CA, USA). Additional quantitave tests of WTD feces included direct fluorescent antibody (DFA) testing to detect *Giardia* spp. and *Cryptosporidium* spp. conducted by the Mississippi Veterinary Research and Diagnostic Laboratory (Pearl, MS, USA).

For statistical analyses, version 9.4 of the SAS System for Windows (©2013 SAS Institute Inc.) was utilized. Differences between each fed and unfed pair for all variables were calculated and Shapiro-Wilk tests were used to determine that all data were non-normally distributed. Therefore, Wilcoxon signed-rank tests were utilized to compare the number of fecal piles and average EPG and OPG of nematodes and coccidia in WTD and raccoon feces. To examine the relationship between coccidia and nematode OPG/EPG and feeder characteristics (feeder type and duration of feeding), generalized linear models (GLMs) using PROC GENMOD were used. A zero-inflated negative binomial distribution was selected for the model due to the number of zeros and the non-normal distribution.

Environmental load was calculated using the mean EPG /OPG of nematodes and coccidia per WTD and raccoon fecal pile, the mean number of fresh feces for these two species per site, and the mean weight of fresh feces for each species (30). A mean weight of 40.8 g was used for raccoon feces (31). To determine mean WTD feces weight, 10 freshly deposited fecal piles were collected from captive WTD at the Mississippi State University deer research facility. The mean weight of these 10 samples was 122.4 g.

## Results

Over the course of the study, 2,133 total fecal piles were counted, of which 288 were deemed fresh enough for fecal egg/oocyte counts. More fecal piles were found at fed sites from WTD (fed-unfed = 8.2 ± 20.8, *p* < 0.0001) and raccoons (2.2 ± 4.2, *p* < 0.0001) (Table 1). Fecal piles from wild pigs (*Sus scrofa)* and rabbits (*Sylvilagus* spp.), were also found at study sites, but they were not included in statistical analyses. Mean number of fresh feceal samples per species per site was <2 (Table 2), but the large number of site pairs allowed us to detect differences in GIP ecology between fed and unfed sites.

**Table 1:** Prevalence, median, and mean number of fecal piles by species found at 79 pairs of fed and unfed sites sampled on 17 properties in Mississippi, USA during May-August of 2019 and 2020.

	**White-tailed deer (** ***Odocoileus virginianus*** **)**		**Raccoon (** ***Procyon lotor*** **)**	
	**Fed**	**Unfed**	**Fed**	**Unfed**
**Overall**				
Median	5	1	1	0
Range	0–264	0–118	0–23	0–6
Mean	14.2	5.9	2.7	0.5
SD	33.8	15.4	4.4	1.1
*P*-value	< 0.001		< 0.001	
Prevalence	85%	71%	58%	24%
**Positives only**				
Median	7	3	2.5	1
Mean	16.7	8.4	4.7	2.1
SD	36.2	17.8	4.9	1.5

The overall means were compared using a Wilcoxon signed rank test on the mean difference between pairs. For each species, positives only was calculated using only sites with at least one scat of that species.

**Table 2:** Estimated environmental load of nematodes and coccidia per site at fed and unfed sites in Mississippi, USA sampled during May-August of 2019 and 2020.

	**Deer**		**Raccoons**		**Total Fed**	**Total Unfed**
	**Fed**	**Unfed**	**Fed**	**Unfed**		
# Fresh feces	1.5	0.8	0.7	0.1	2.2	0.9
Coccidia environmental load	70,135.2	37,405.4	80,396.4	11,485.2	150,531.6	48,890.6
Nematode environmental load	21,848.4	11,652.5	7,568.4	1,081.2	29,416.8	12,733.7

Feces were identified as either white-tailed deer (*Odocoileus virginianus*) or raccoon (*Procyon lotor*). Environmental load was calculated by multiplying the mean number of fresh fecal piles per species per site, the average eggs per gram (EPG) feces per species (Table 3), and the average weight of a fresh fecal pile for deer (122.4 g) and raccoons (40.1 g). Totals for fed and unfed is the sum of the two species.

Gastrointestinal parasite loads for individual fecal samples did not differ between fed and unfed sites for WTD or raccoons (Table 3). Nematodes were not affected by feeder type or feeding duration (Table 4). For coccidia, there was also no effect of feeding duration. However, WTD parasite loads within individual fecal samples were greater around spin feeders (Table 4), with 217% more coccidia oocysts in WTD feces at spin feeders than at gravity and 650% more than at trough on average.

**Table 3:** Summary statistics for coccidia oocysts per gram (OPG) and nematode eggs per gram (EPG) in scat by host species collected during May-August of 2019 and 2020 at 79 site pairs on 17 properties in Mississippi, USA.

	**Coccidia**		**Nematodes**	
	**Deer**	**Raccoons**	**Deer**	**Raccoons**
N	178	61	178	61
**Overall**				
Median	0	520	40	60
Range	0–39,540	0–26,060	0–2,680	0–5,040
Mean	382	2,815	119	265
SD	3,016	4,881	248	817
Prevalence	29%	80%	72%	79%
**Positives only**				
Median	0	580	80	60
Mean	507	3,013	158	284
SD	3,471	4,992	274	842

Fresh feces were from white-tailed deer (Odocoileus virginianus) and raccoons (Procyon lotor). For each species, positives only were calculated using only feces in which at least one GIP was detected. Between fed and unfed sites, no GIP by species differed by Wilcoxon signed-rank tests, nor did their prevalence, by chi-squared tests.

**Table 4:** Generalized linear model-generated coefficients for the effects of feeder type (gravity and spin relative to trough) and duration of feeding (5–10 years, 1–4 years) on the infection intensity of feces with coccidia and nematodes collected in Mississippi, USA during May-August of 2019 and 2020.

**Variable**	**Estimate**	**Variance SE**	**95% Confidence Interval**
**Nematodes**			
Feeder type (gravity)	−0.04	0.92	−1.84, 1.75
Feeder type (spin)	−0.01	0.62	−1.22, 1.20
Feeder duration (5–10 years)	−0.63	0.61	−1.83, 0.56
**Coccidia**			
Feeder type (gravity)	−1.43	2.02	−5.39, 2.54
Feeder type (spin)	2.98	1.32	0.40, 5.56
Feeder duration (5–10 years)	1.69	1.37	−1.00, 4.38

Environmental parasite load varied more between paired sites for coccidia than nematodes, but in both cases were greater at fed sites than unfed sites (Table 2). Despite no difference in the average GIP load for each individual fecal pile, the increased number of feces at fed sites led to 231 and 308% greater environmental loads of nematodes and coccidia, respectively, compared to unfed sites.

Human visitation differed in prevalence (*p* < 0.01) and average daily visits (*p* < 0.01) at fed (54%, 0.2 ± 0.3 visits/day) and unfed sites (29%, 0.1 ± 0.2 visits/day). In addition to humans and wildlife, domestic dogs (*Canis familiaris*) were also documented more at fed sites than unfed sites (11 vs. 2 visits), though the sample size was too low for statistical comparison.

**Figure 3:**
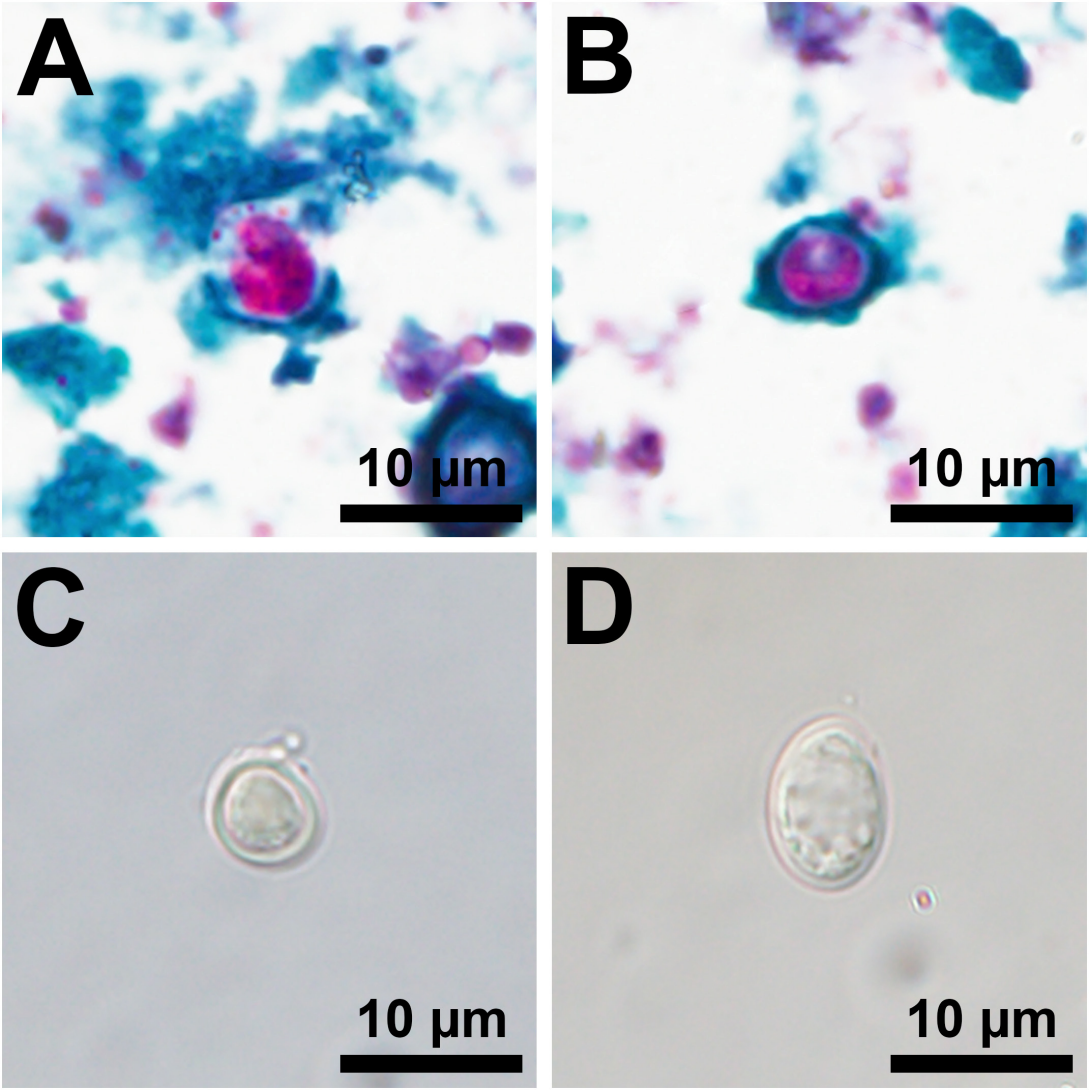
Photomicrographs of acid-fast stained oocysts consistent with a Cryptosporidium sp. **(A,B)** and isolations by fecal floatation using Sheather's saturated sugar solution of an unstained oocyst consistent with a Cryptosporidium sp. **(C)** and an unstained cyst consistent with a Giardia sp. **(D)**.

Sixty-two sites had at least one fresh WTD scat and composite samples from each of these sites were tested for *Giardia* spp. and *Cryptosporidium* spp. Two composite samples, one fed and one unfed site on different properties, were positive for both *Giardia* spp. and *Cryptosporidium* spp. (Figure 3). Due to a low detection rate, statistical analyses could not be performed. The assemblage/species of *Giardia* spp. and *Cryptosporidium* spp. was not detected in this study; therefore, the zoonotic potential of the parasites detected is unknown. Two of 61 individual, fresh raccoon scats contained eggs morphologically suggestive of a roundworm species, but neither were positive for *B. procyonis* by RT-PCR.

## Discussion

For many individuals who supplementally feed wildlife, the goal of the feeding program is to improve wildlife health (2). However, these results suggest supplemental feeding increases environmental load of gastrointestinal parasites, which may undermine nutritional gains from feeding (32) and risk increasing disease transmission. Previous studies have reported similar results, such as increased GIPs with presence of supplemental feeding (6, 7) and greater host density (33).

It is unsurprising that fecal EPG counts in this study did not differ by feeding status given that paired fed and unfed sites averaged only 147 m apart. Not all WTD in a population will visit feeders (26), but the same individual could visit both fed and unfed sites within a pair since bucks travel 6 km/day on average (34). Similarly, average raccoon home ranges in Mississippi range from 1.3 to 2.8 km^2^ (35), demonstrating that the same individual could travel between fed and unfed sites. The difficulty in finding a large sample size of ecologically-equivalent unfed sites that were also spatially independent from feeder locations prevented us from using populations with no access to feed as past research has (5, 36). We were also limited in how far away we could establish unfed sites while matching the ecological and management traits of fed sites.

Environmental load calculations demonstrated one potential effect of concentrating wildlife at a supplemental feeding site. The increased environmental load of GIPs that are transmitted through fecal oral or direct contact with larva in the environment (e.g., coccidia and nematodes, respectively) suggests supplemental feeding may affect disease transmission. The increased visitation to supplemental feeding sites by humans, wildlife, and possibly domestic pets, coupled with greater environmental parasite loads, may promote the transmission of zoonotic diseases such as *Cryptosporidium parvum* or *B. procyonis*, both of which have been detected in many wildlife species, humans, and domestic animals (9, 10, 13). While these specific zoonotic parasites were not detected in this study, they have been detected in numerous other wildlife species that would likely be attracted to supplemental feedings sites (13, 37). This suggests that supplemental feeding could affect transmission risk of zoonotic parasitic diseases in geographic areas where they are present. This increased risk, coupled with increased human visitation to feeding sites, could be a public health concern.

Interestingly, there was an effect of feeder type with increased coccidia in feces around spin feeders, where feed is consumed off the ground, compared to gravity and trough feeders, where feed is designed to be consumed directly from the feeder (Figure 2). This suggests an elevated transmission risk from feeding practices that distribute food directly onto the ground, which is plausible particularly for parasites with fecal-oral transmission, such as coccidia (24).

Despite our hypothesis that gastrointestinal parasitism would increase over time at feeding sites, there was no effect of time for either nematodes or coccidia. It was expected that GIPs would increase with long-term feeding because the proportion of WTD using feeders increased over time in Hubert and others' study (26). However, these results were over the first 2 years of feeding whereas this study compared 1–4 years of feeding to 5–10 years of feeding. At this scale, there may not be meaningful differences in the proportion of a WTD population using feeders. Alternatively other wildlife use may not differ in time in the same way and so mitigate any effect of changing WTD use.

*Giardia* spp. and *Cryptosporidium* spp. were detected in only 3.2% of samples, which is similar to previously reported surveillance data for Mississippi WTD (*Giardia* spp. and *Cryptosporidium* spp. prevalence of 1.1 and 5.0%, respectively; (38). *Giardia* spp. is typically transmitted through water sources, but it can also be through direct contact with feces (39). Future studies with more samples that determine *Giardia* spp. and *Cryptosporidum* spp. species/assemblage should be conducted to determine the effects of supplemental wildlife feeding on specifically zoonotic species/assemblages. To the authors' knowledge, *Baylisascaris procyonis* has still not been found in Mississippi (13). This study should be repeated in a geographic area with high *B. procyonis* prevalence, such as Texas, to determine the effects of supplemental feeding on this parasite of high public health importance. Sampling GIPs from feces found in the field has limitations. First, freshness of collected feces was unknown, unlike studies which used freshly-deposited samples or samples collected directly from the animal (38, 40). Additionally, for *Giardia*, cysts are excreted sporadically during infection (41), so prevalence may have been under-estimated.

In conclusion, supplemental feeding, like other actions that concentrate animals, increases GIP environmental load which may increase disease transmission risk to wildlife, domestic pets, and humans. This effect may be mitigated by using feeders that keep feed off the ground, like gravity and trough feeders. Future research can further elucidate the relationship between feeding and GIPs by comparing isolated populations with and without feeding.

## Data availability statement

The raw data supporting the conclusions of this article will be made available by the authors, without undue reservation.

## Author contributions

MH contributed to project design, data collection, statistical analysis, and drafting the manuscript. SD contributed to project design and implementation. WCB contributed to project design, data collection, and drafting the manuscript. BS contributed to project design, implementation, and statistical analysis. All authors contributed to manuscript review and revision. All authors contributed to the article and approved the submitted version.

## Funding

This publication is a contribution of the Forest and Wildlife Research Center, Mississippi State University and was supported by the Mississippi Department of Wildlife, Fisheries, and Parks using US Fish and Wildlife Service (Pittman-Robertson) Federal Aid in Wildlife Restoration funding.

## Conflict of interest

The authors declare that the research was conducted in the absence of any commercial or financial relationships that could be construed as a potential conflict of interest.

## Publisher's note

All claims expressed in this article are solely those of the authors and do not necessarily represent those of their affiliated organizations, or those of the publisher, the editors and the reviewers. Any product that may be evaluated in this article, or claim that may be made by its manufacturer, is not guaranteed or endorsed by the publisher.
